# Supplementary material for: MesoGraph: Automatic profiling of mesothelioma subtypes from histological images
Source: Cell Rep Med. 2023 Oct 9;4(10):101226. doi: 10.1016/j.xcrm.2023.101226 (PMC10591053; doi:10.1016/j.xcrm.2023.101226)
Supplement: Document S1. Figures S1–S4 [file mmc1.pdf]

Cell Reports Medicine, Volume *4*

Supplemental information

MesoGraph: Automatic profiling of mesothelioma subtypes from histological images

Mark Eastwood, Heba Sailem, Silviu Tudor Marc, Xiaohong Gao, Judith Offman, Emmanouil Karteris, Angeles Montero Fernandez, Danny Jonigk, William Cookson, Miriam Moffatt, Sanjay Popat, Fayyaz Minhas, and Jan Lukas Robertus

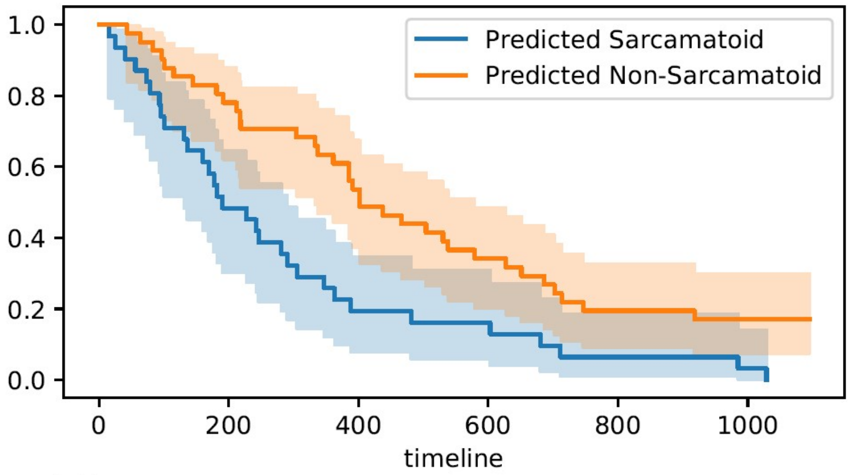

**Predicted Non-Sarcamatoid**						
At risk	41	32	22	14	8	7
Censored	0	0	0	0	0	0
Events	0	9	19	27	33	34
**Predicted Sarcamatoid**						
At risk	31	15	6	5	2	1
Censored	0	0	0	0	0	0
Events	0	16	25	26	29	30

Figure 1: Kaplan-Meier curves for survival prediction. Related to Figure 5. Shows curves when stratifying by model output score with data right censored at 3 years.

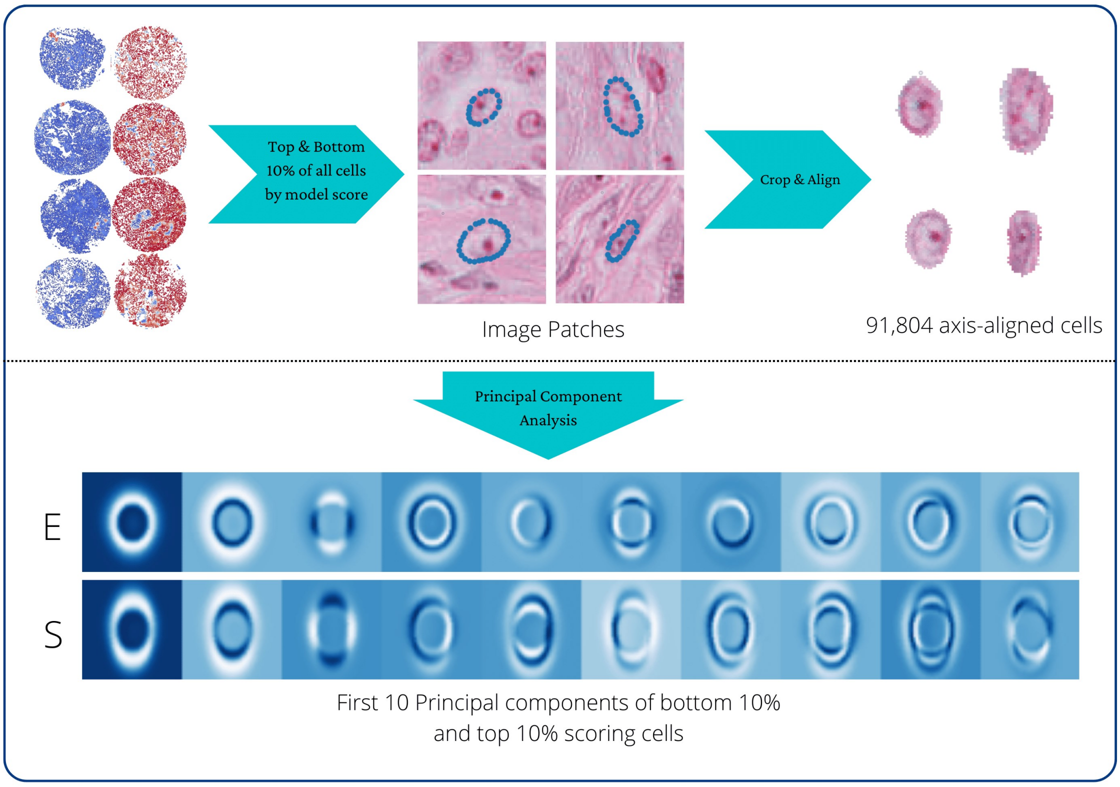

Figure 2: Principal component analysis on the top & bottom 10% of cells by model score. Related to Figure 4. Top and bottom scoring cell populations were oriented so that their major axes are oriented in the same direction, and cropped to remove as much background as possible. The resulting PCA components show highly sarcomatoid-scoring cells align with known morphological features of sarcomatoid subtype.

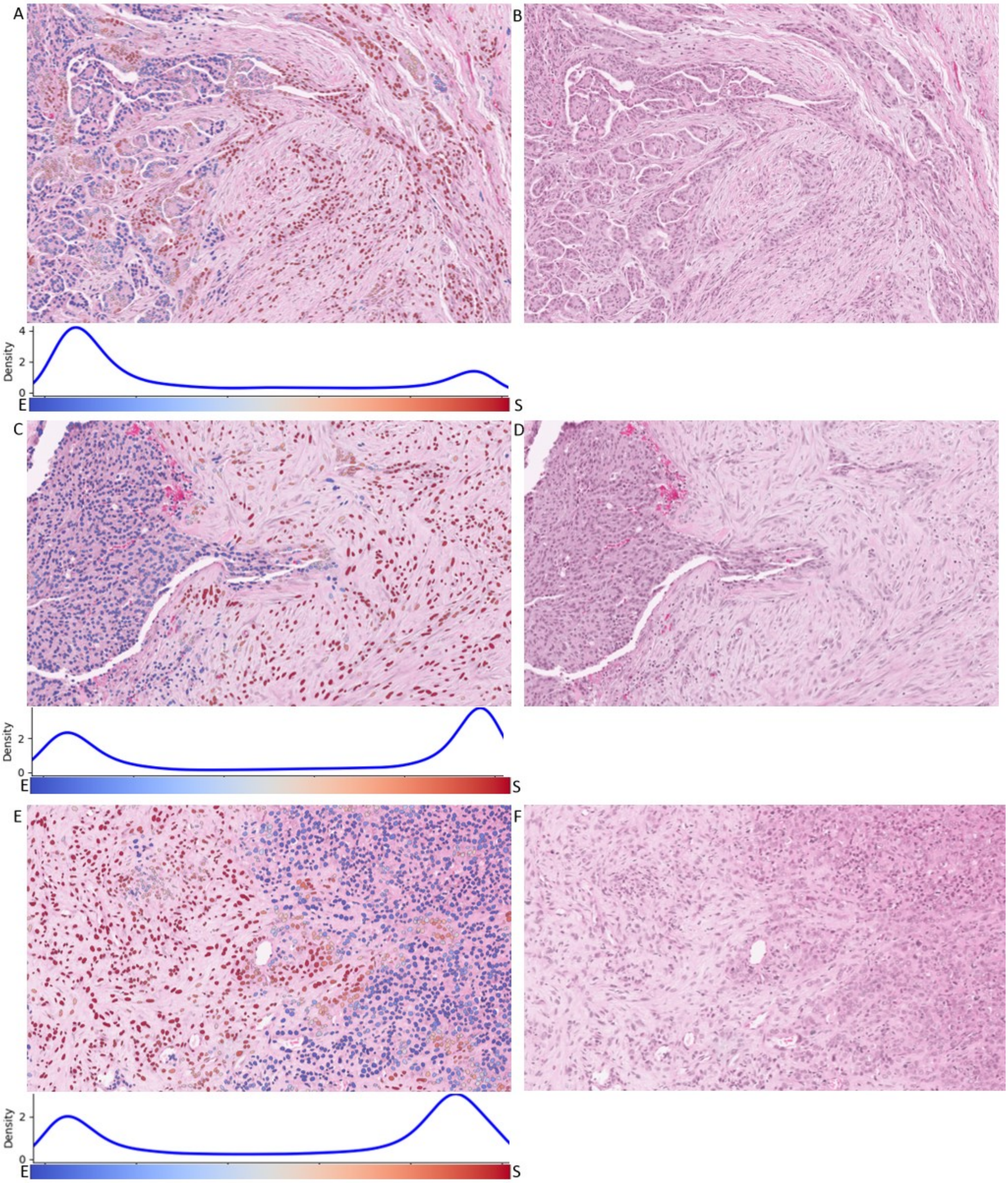

Figure 3: Model results on biphasic TCGA ROIs. Related to Figure 1. Illustrates the ability of MesoGraph to quantify regions of differing histological subtype. On the left in panels A, C and E are the rois overlaid with model output, and on the right in panels B, D and F are the raw ROI images. Distinct regions of sarcomatoid and non-sarcomatoid component can be seen defined by the model. The 'Mesogram' plot below each overlaid image shows the distribution of cell scores in the ROI.

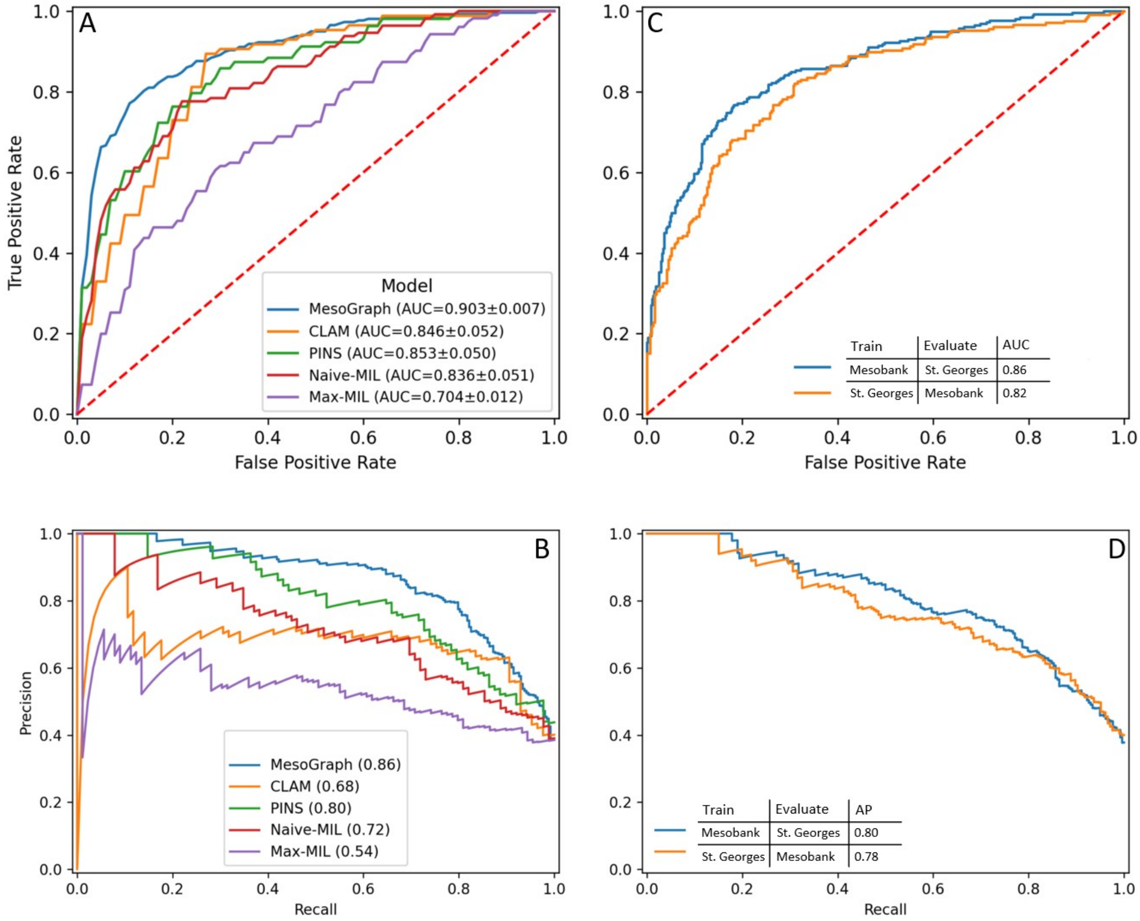

Figure 4: AUROC curves for experiments performed. Related to Table 1. A) AUROC for each model. B) Model average precision. C) AUROC on cross-cohort evaluation. D) Average precision on cross-cohort evaluation. Our model outperforms other models tested, and from C and D we see good generalization to external validation data.
