# Supplementary material for: MesoGraph: Automatic profiling of mesothelioma subtypes from histological images
Source: Cell Rep Med. 2023 Oct 9;4(10):101226. doi: 10.1016/j.xcrm.2023.101226 (PMC10591053; doi:10.1016/j.xcrm.2023.101226)
Supplement: Document S2. Article plus supplemental information [file mmc2.pdf]

Cell Reports
Medicine

Article

MesoGraph: Automatic profiling of mesothelioma subtypes from histological images

Graphical abstract

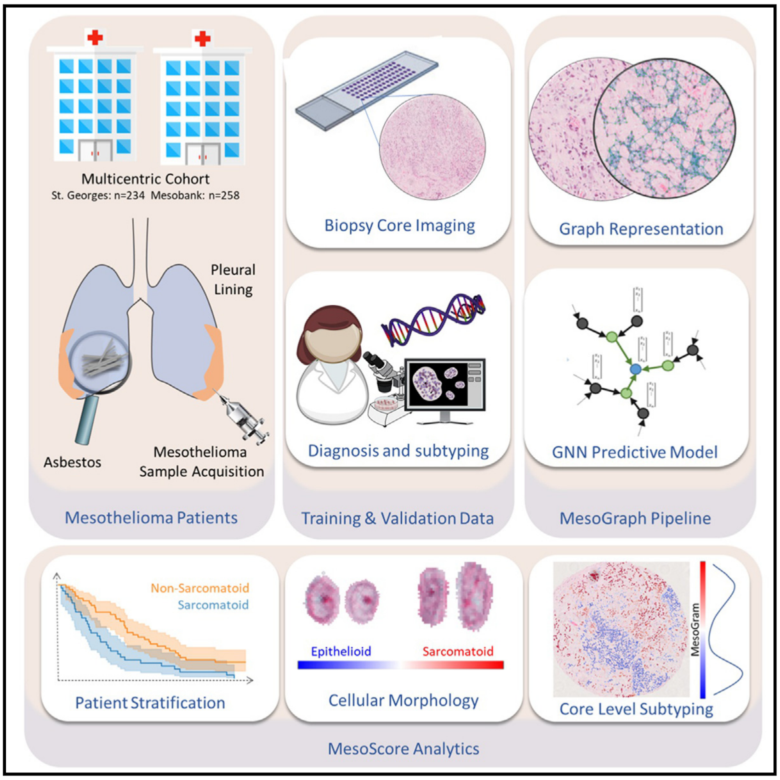

Highlights

- GNN capable of scoring regions of tissue according to its sarcomatoid association
- Morphological analysis agrees with known characteristics of subtypes
- AUROC of 0.90 in subtype prediction task
- Model score shown to be associated with survival with hazard ratio 2.30

Authors

Mark Eastwood, Heba Sailem, Silviu Tudor Marc, ..., Sanjay Popat, Fayyaz Minhas, Jan Lukas Robertus

Correspondence

mark.eastwood@warwick.ac.uk

In brief

Eastwood et al. introduce MesoGraph, a graph neural network model for the profiling of mesothelioma subtype from tissue images. A quantitative measure of the prevalence of sarcomatoid regions in a mesothelioma sample could allow a more accurate and less subjective assessment of tissue samples.

Eastwood et al., 2023, Cell Reports Medicine *4*, 101226
October 17, 2023 © 2023 The Authors.
https://doi.org/10.1016/j.xcrm.2023.101226

CellPress

Cell Reports Medicine

CellPress
OPEN ACCESS

Article

MesoGraph: Automatic profiling of mesothelioma subtypes from histological images

Mark Eastwood,[1,13,*] Heba Sailem,[2,4] Silviu Tudor Marc,[3] Xiaohong Gao,[3] Judith Offman,[4,5] Emmanouil Karteris,[6] Angeles Montero Fernandez,[7] Danny Jonigk,[8,9] William Cookson,[10] Miriam Moffatt,[10] Sanjay Popat,[10] Fayyaz Minhas,[1,11,12] and Jan Lukas Robertus[10,12]

[1]Tissue Image Analytics Center, University of Warwick, Coventry, UK
[2]Institute of Biomedical Engineering, University of Oxford, Oxford, UK
[3]Department of Computer Science, University of Middlesex, London, UK
[4]Kings College London, London, UK
[5]Wolfson Institute of Population Health, Queen Mary University of London, London, UK
[6]College of Health, Medicine and Life Sciences, Brunel University London, London, UK
[7]Manchester University, Manchester, UK
[8]German Center for Lung Research (DZL), BREATH, Hanover, Germany
[9]Institute of Pathology, Medical Faculty of RWTH Aachen University, Aachen, Germany
[10]National Heart and Lung Institute, Imperial College London, London, UK
[11]Warwick Cancer Research Centre, University of Warwick, Coventry, UK
[12]These authors contributed equally
[13]Lead contact
*Correspondence: mark.eastwood@warwick.ac.uk
https://doi.org/10.1016/j.xcrm.2023.101226

SUMMARY

Mesothelioma is classified into three histological subtypes, epithelioid, sarcomatoid, and biphasic, according to the relative proportions of epithelioid and sarcomatoid tumor cells present. Current guidelines recommend that the sarcomatoid component of each mesothelioma is quantified, as a higher percentage of sarcomatoid pattern in biphasic mesothelioma shows poorer prognosis. In this work, we develop a dual-task graph neural network (GNN) architecture with ranking loss to learn a model capable of scoring regions of tissue down to cellular resolution. This allows quantitative profiling of a tumor sample according to the aggregate sarcomatoid association score. Tissue is represented by a cell graph with both cell-level morphological and regional features. We use an external multicentric test set from Mesobank, on which we demonstrate the predictive performance of our model. We additionally validate our model predictions through an analysis of the typical morphological features of cells according to their predicted score.

INTRODUCTION

Malignant Mesothelioma (MM) is an aggressive cancer of malignant mesothelial cells of the pleural lining, primarily associated with asbestos exposure.[1] It has a poor prognosis with less than 10% 5 year survival rates due to late diagnosis.[2,3] It has a long latency period from initial exposure to eventual carcinogenesis and is difficult to diagnose due to its non-specific clinical manifestations. MM is classified into 3 subtypes,[4] epithelioid, biphasic, and sarcomatoid mesotheliomas (EM, BM, and SM, respectively), with BM characterized by a mix of epithelioid and sarcomatoid components. The histological subtype of mesothelioma is essential for prognosis and clinical decisions on treatment pathways for patients.[5] Stratification of a given sample into a particular subtype informs treatment and can help gain a more in-depth understanding of disease pathology and outcome. The benefit of surgical treatment has prognostic implications for EM with a median survival of 19 months but less so for SM and BM, with respective median survivals of 4 and 12 months after surgical treatment.[6]

EM is characterized by malignant cells that are cytologically round with varying gradings of atypia. SM cells are generally recognized as malignant elongated spindle cells[7] and are associated with worse prognosis in comparison with EM. SM cells may also include transitional features that are intermediate between epithelioid and sarcomatoid. Although transitional cells are now classified under SM, their presence is associated with worse prognosis.[8]

While the distinction of these three histological subtypes of MM is crucial to patient treatment, management, and prognosis, it is challenging to differentiate EM, SM, and BM through visual analysis. Currently, there are no clear guidelines on how to perform this stratification in an objective and reproducible manner.[9] Furthermore, even though mesotheliomas are divided into these three broad categories, in reality, there is a continuous spectrum from EM to SM dependent upon the relative proportion

This is an open access article under the CC BY license (http://creativecommons.org/licenses/by/4.0/).

of epithelioid and sarcomatoid cells in a given sample. As a consequence, existing approaches are unable to objectively quantify where on this spectrum a given sample falls based on profiling of cellular morphological patterns in it.

A number of deep learning methods for analyzing mesothelioma images have been developed recently. For example, SpindleMesoNet[10] can separate malignant SM from benign spindle cell mesothelial proliferations. A recent approach for survival prediction of patients with MM called MesoNet[8] uses a multiple instance learning (MIL) solver originally developed for computer vision applications[11] and classification of lymph node metastases.[12] However, automated subtyping of mesothelioma from hematoxylin and eosin (H&E)-stained tissue sections remains an open problem.

One challenge in the characterization of mesothelioma is that pathologist-assigned ground-truth labels of mesothelioma subtypes are typically available only at the case level, as it is very difficult for pathologists to associate tumor microenvironment or cellular morphometric patterns with image- or case-level labels in an objective manner. Moreover, it can be very time consuming to obtain detailed cellular or regional annotations, and those annotations may not be very reliable due to significant inter- and intra-observer variation.

The aim of this study was to develop a graph neural network (GNN) approach to predict subtypes of mesothelioma in an MIL setting. This was achieved considering tissue microarray (TMA) cores as bags and individual cells as instances. On these, we have built a weakly supervised machine learning model to characterize mesothelioma subtypes using only case-level labels in its training. The proposed approach can generate a quantitative assessment of where the sample stands in terms of the aforementioned epithelioid to sarcomatoid spectrum, enabling pathologists to perform a more in-depth characterization of tumor samples. An overview of the approach presented in the article can be found in Figure 1.

RESULTS

We have developed a custom GNN-based pipeline called MesoGraph that can predict mesothelioma subtypes using H&E-stained tissue images. MesoGraph uses pathologist-assigned case-level labels without any cellular or regional annotations in its training. The proposed approach models each cell in a given sample as a node in the graph, which is connected to neighboring cells. Each node is associated with various features, which can be broadly classified into four types: (1) nuclear morphometric features, (2) stain intensity features of nuclear and cytoplasmic components of the cell, (3) cellular counts in the neighborhood of node, and (4) deep neural network and Haralick-based texture features. For a given test sample, it generates two probability scores (collectively called MesoScore) representing the probabilities of the sample being epithelioid or sarcomatoid. As a BM tumor is composed of both epithelioid and sarcomatoid components, the two outputs in MesoScore allow precise quantification of the two components in the sample. In addition to predicting mesothelioma subtype, MesoGraph also generates cell-level quantitative scores representing the association of each cell with the mesothelial subtype of the given sample. MesoGraph has been trained and independently validated on two datasets: St. George's Hospital (SGH; n = 234) and the multicentric Mesobank (MB) collection (n = 258).

In this section, we present the results of the proposed method in terms of its predictive performance in comparison to existing approaches, as well as its ability to identify histological features and morphological characteristics of cells associated with different subtypes of mesothelioma. We also demonstrate the ability of the proposed approach to stratify patients with mesothelioma based on their expected survival.

Predictive performance

Test results from the MesoGraph pipeline for both MB and SGH datasets are shown in Figure S4 and Table 1. Here, the receiver operating characteristic (ROC) curve is obtained by considering both sarcomatoid and biphasic samples as the positive class, whereas the epithelioid samples are associated with the negative label. As can be seen from these results, the proposed approach offers high predictive quality over both cross-validation and independent testing in comparison to other existing approaches. In the table, PINS refers to the positive instance sampling patch-based MIL approach as detailed in Eastwood et al.,[13] whereas CLAM is the clustering-constrained attention MIL method described in Lu et al.,[14] a deep-learning-based weakly supervised method that uses attention in combination with clustering-based constraints to identify the most predictive areas of the image. Max-MIL and naive-MIL are simple patch-based baseline MIL methods detailed further in the STAR Methods Model performance and evaluation. As can be seen from Table 1, the max-based MIL strategy performs poorly. This is likely due to the relatively small size of the training dataset, as learning only on the maximally scoring instance per bag exacerbates this. Naive-MIL performs surprisingly well. This may be due to the relatively high proportion of positive instances that are expected to be present in many of the positive bags (for example, a sarcomatoid core should contain mostly positive instances). This makes the implicit assumption this model makes, namely that all instances share the label of the bag, less wrong for this dataset compared with other MIL tasks. PINS and CLAM, as two patch-based methods with a mechanism for focusing on the most relevant region of an image, perform similarly with solid performance. However, as patch-based methods, the spatial resolution of the prediction maps they can provide is lower than that of our cell-graph-based model.

Our model outperforms other models tested, achieving an internal cross-validation performance of 0.90 ± 0.01. It performs particularly well in terms of its average precision (AP) of 0.86 ± 0.02, indicating that its performance on the positive class (which is the minority class) is very good. While performance drops slightly on the external validation set, an area under the ROC (AUROC) of 0.86 and an AP of 0.8 as seen in Figures S4C and S4D shows that these results generalize well. We attribute the performance improvements achieved by our model firstly to the cell graph representation, with cells and their morphological features as instances, which is far more natural than an arbitrary division into patch instances, and secondly, to our formulation of the learning as a dual-task problem with a ranking loss. The ranking loss acknowledges the ordering we know exists between EM, BM, and SM cores in terms of how much of a sarcomatoid

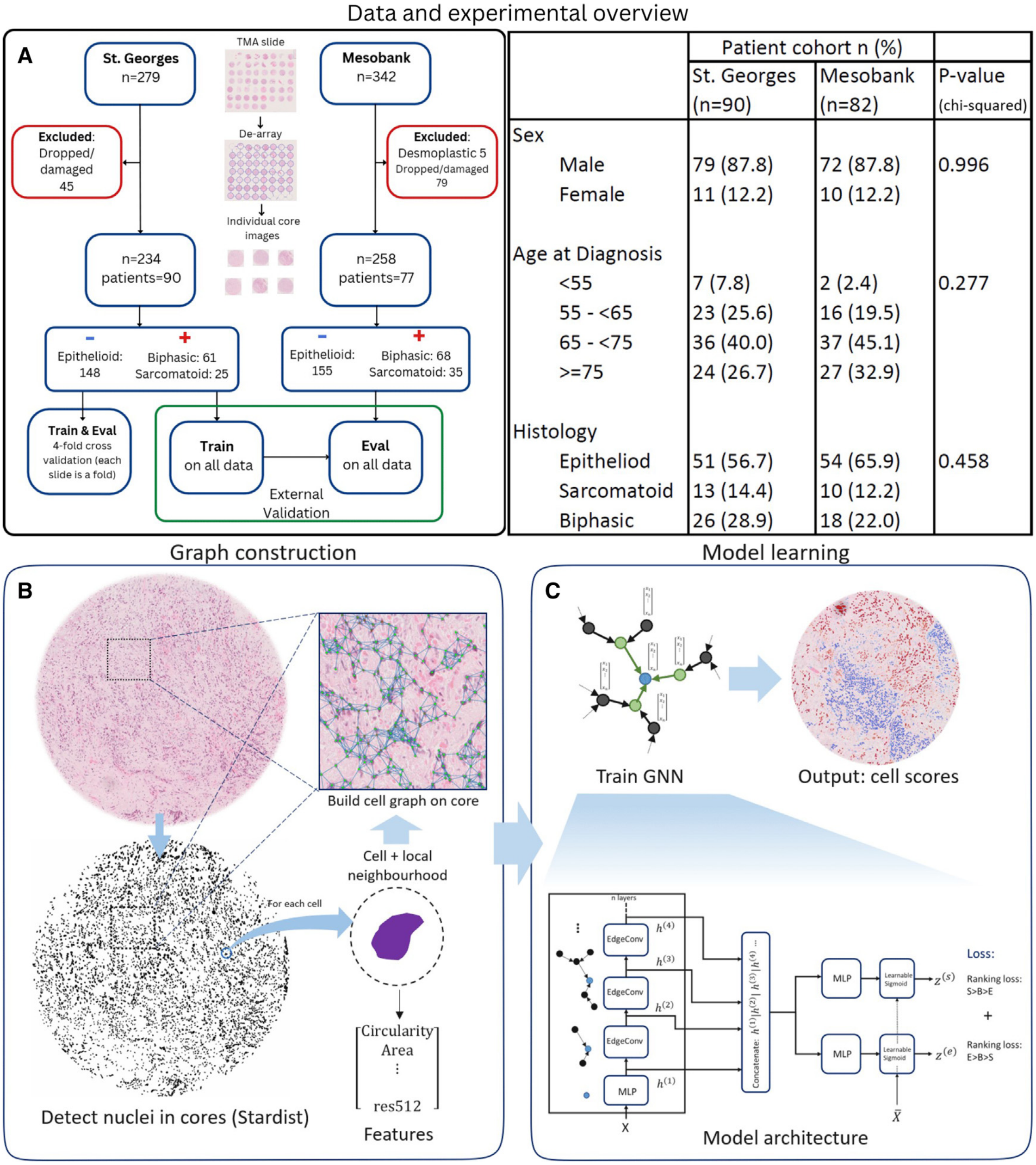

	Patient cohort n (%)		
	St. Georges (n=90)	Mesobank (n=82)	P-value (chi-squared)
Sex			
Male	79 (87.8)	72 (87.8)	0.996
Female	11 (12.2)	10 (12.2)	
Age at Diagnosis			
<55	7 (7.8)	2 (2.4)	0.277
55 - <65	23 (25.6)	16 (19.5)	
65 - <75	36 (40.0)	37 (45.1)	
>=75	24 (26.7)	27 (32.9)	
Histology			
Epitheliod	51 (56.7)	54 (65.9)	0.458
Sarcomatoid	13 (14.4)	10 (12.2)	
Biphasic	26 (28.9)	18 (22.0)	

Figure 1. Overview of the study, model, and experimental design

(A) Data and experimental design. TMA slides were de-arrayed into individual images, and images of cores that were dropped or particularly badly damaged were excluded. The model is trained on the St. George's cohort and validated both internally and on the external Mesobank cohort.

(B and C) Steps to represent a TMA core as a graph, from cell detection, through extraction of morphological and local neighborhood features, to the construction of the cell graphs upon which our model will be trained. In (C) is the proposed MesoGraph GNN architecture. Deeper layers incorporate information from larger neighborhoods. By concatenating layer representations, we allow the model to use information at multiple scales.

component is present, and the dual-task formulation allows the possibility that some regions of tissue may not be strongly associated with either a sarcomatoid or an epithelioid core label.

Visualization of model output

The output of our model can be visualized in a zoomable, interactive graphical user interface (GUI) we have developed. A demo of results from our model can be found at https://mesograph.dcs.warwick.ac.uk. Examples of the cell-level scoring output by the model are shown in Figure 2. Further examples of model output on biphasic whole-slide image (WSI) samples from The Cancer Genome Atlas (TCGA) dataset, illustrating the ability of our model to define regions of differing histological subtype, can be found in Figure S3. For each cell in a given sample, the proposed model

Table 1. Summary of results of models evaluated on a primary dataset (SGH)

Metric	AUROC	Avg. precision	Sensitivity	Specificity
Max-MIL	0.70 ± 0.01	0.54 ± 0.12	0.54 ± 0.07	0.73 ± 0.09
Naive-MIL	0.84 ± 0.05	0.72 ± 0.11	0.72 ± 0.08	0.71 ± 0.1
PINS[13]	0.85 ± 0.05	0.80 ± 0.07	0.82 ± 0.1	0.71 ± 0.13
CLAM[14]	0.85 ± 0.07	0.74 ± 0.11	0.75 ± 0.11	0.77 ± 0.02
MesoGraph (ours)	0.90 ± 0.007	0.86 ± 0.02	0.88 ± 0.015	0.72 ± 0.01

Mean ± SD is shown for each metric. Avg, average.

generates two prediction scores signifying the probability of the cell being associated with a sarcomatoid or an epithelioid label. These scores can be combined and visualized in a colormap showing cells that are associated with epithelioid (blue) and sarcomatoid (red) subtypes, as well as in a histogram (called MesoGram) showing the relative distributions of epithelioid and sarcomatoid components.

From the zoomed-in masks, we can see that the model can distinguish between regions with typical rounded morphology of the epithelioid subtype and the more elongated morphology displayed in sarcomatoid regions. The MesoGram plots of most samples tend to be bimodal to some extent, with epithelioid and sarcomatoid cores more heavily skewed toward low and high scores, respectively. This continuum of distribution between sarcomatoid and epithelioid is demonstrated further in Figure 3, where thumbnails of model output on all cores are shown, grouped by subtype and ordered within each subtype by model score. This ability to give a more precise, fine-grained characterization of a tumor beyond the current three poorly defined and subjective subtypes is a strength of our approach.

Explainability of model predictions

To gain an understanding of the predictions generated by the proposed approach, we have applied GNNExplainer[15] to the trained GNN model. This allows us to understand what node-level features are contributing to the prediction of a given sample for each subtype. The top 10 features identified in this analysis are shown in Figure 4.

The most important feature overall is the circularity, which confirms the expected distinction between the rounder morphology of the epithelioid subtype compared with the more spindle-shaped sarcomatoid morphology. There are also a number of features describing the intensity and texture in the eosin channel around the nucleus. Looking at the feature importances on specific subtypes, the resnet features are most useful on epithelioid cores. Circularity is specifically important in epithelioid and sarcomatoid subtypes, as they tend to be composed of more homogeneous populations and therefore are expected to contain mostly either rounded or more elongated cells. In both biphasic and sarcomatoid cores, nearby detection counts seem to be an important feature. This may reflect a tendency for non-epithelioid tumors to display a slightly more spread out and disorganized cell distribution. We also notice that the importance of most of the top features has far more spread when considering epithelioid cores, indicating that a wide variety of features can contribute to an epithelioid score, with few features being universally important across all epithelioid cores.

To determine the separation between classes based on top-scoring features, in the bottom half of Figures 5C and 5D, we plotted the prediction of each core against the assigned label by a pathologist. While epithelioid cores and sarcomatoid cores are mostly well separated, we observe that there can be overlap between some of the cases in terms of morphology. We also observe that biphasic cores are not very distinct from sarcomatoid cores.

Characterization of cellular morphologies

Pathologists assess cell morphology when diagnosing and scoring mesothelioma tumors. Therefore, we sought to investigate differences in nuclear morphology between mesothelioma tumors with different diagnoses. We focused on key features assessed qualitatively by pathologists including nuclear area, elongation (width and length) and nucleus shape regularity as measured by both circularity (how close it is to a circular shape), and solidity (which reflects overall concavity of a shape). Interestingly, in Figure 5, we found that sarcomatoid tumors tend to have larger nuclei on average compared with epithelioid tumors. As expected, these nuclei are more elongated and have less circularly shaped. For almost all features, measures of nucleus shape in biphasic tumors fall in between epithelioid and sarcomatoid tumors. These results already confirm that our image analysis pipeline reflects inherent differences between these types even when only considering the average measures of each tumor, which is consistent with pathological features. This motivates the development of more sophisticated AI methods to detect these differences.

Next, we investigated the extent of variability in morphological measurements across different tumor types. We measured the standard deviation of cell features for each single tumor core as a proxy of heterogeneity. We found that sarcomatoid tumors exhibit higher morphological heterogeneity in all nuclear features. These analyses motivate the investigation of single-cell phenotypes to identify the most relevant subpopulations.

We can gain further insight into the differences in morphology that the model is associating with each subtype by looking at the principal components of cells assigned the highest and lowest scores (i.e., most and least likely to be associated with a sarcomatoid core, respectively).

Comparing the first principal component for each subtype in Figure S2, we can see that the model has learned to assign a higher score to cells with a more elongated morphology. This reflects a known distinguishing feature of the sarcomatoid morphology, validating our model scoring. This is further illustrated in the scatterplot in the top half of Figure 5, where we

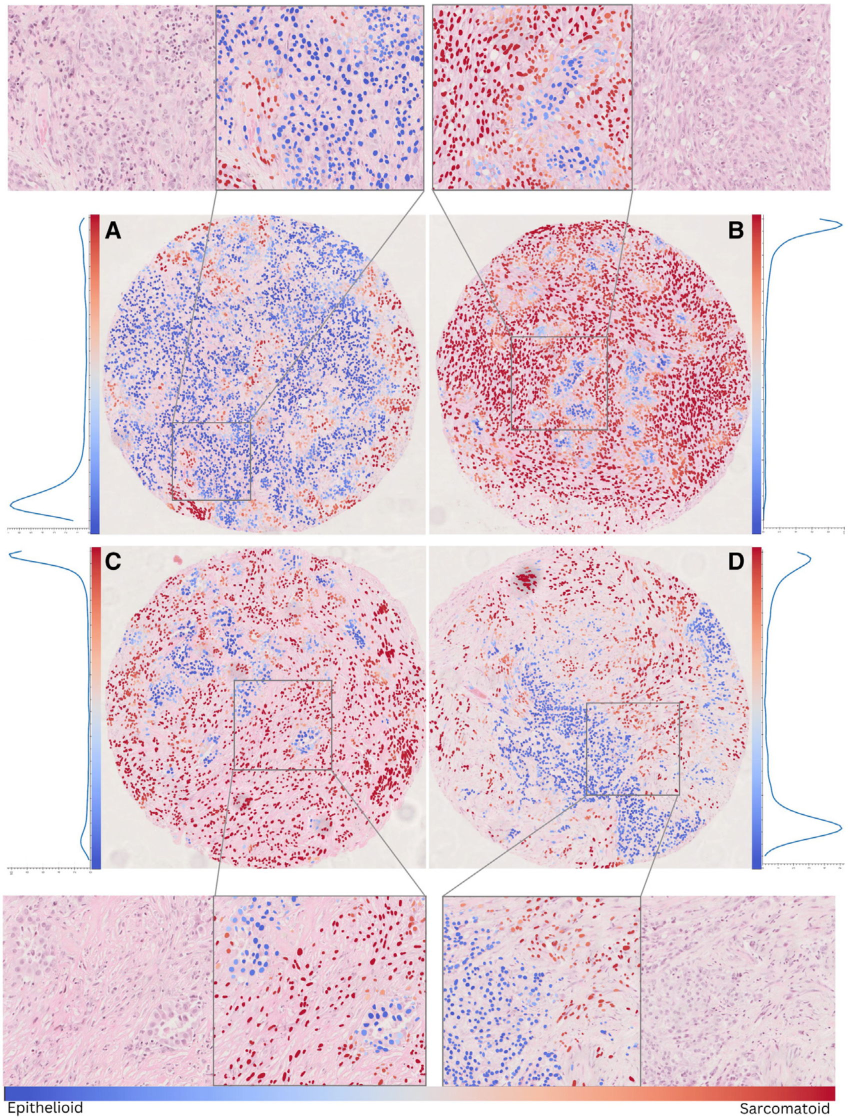

Figure 2. Examples of model visualization on a selection of TMA cores

Each core is shown together with a zoomed-in view showing the differences in morphology between regions and a plot showing the distribution of node scores in that core.

(A and B) Epithelioid core. (A) We see a predominantly low-scoring core with only a few small regions displaying slightly more sarcomatoid features. Conversely, in (B), a sarcomatoid core, nodes are predominantly high scoring.

(C and D) Biphasic cores. In each core, we see a bimodal distribution of scores, particularly pronounced in core (D). The zoomed-in regions show a distinct difference in morphology between high- and low-scoring regions, with rounder cells seen in lower-scoring regions and a more elongated morphology and less structured cell organization in higher-scoring regions.

show a supervised uniform manifold approximation and projection (UMAP)[16] of the principal components of high- and low-scoring cells. The supervisory signal is provided by the output of our model as a binary label of whether it is in the top or the bottom 10% of cells by score. Each point in the map represents a cell, colored red if it is in the top 10% of model scores or blue if in the bottom 10%. UMAP attempts to learn an embedding in which examples with similar features are closer together. Thus, by looking at groups of cells in this map, we can understand how high- and low-scoring cell populations look. From Figure 5's sarcomatoid groups B and E, we can see that elongated cells are scored highly sarcomatoid, as are groups C and D, which show large, irregular cells. Cells scored in the bottom 10% tend to be much smaller, as can be seen in groups F, H, I, and J. They also are rounder and more regular in their shape. As can be seen comparing epithelioid group G with sarcomatoid group C, while large cells may also be scored as epithelioid, they have a round shape with less texture to the staining.

Survival analysis using MesoScores

Survival analysis using Kaplan-Meier plots are shown in Figure 6. Patients were divided into two groups based on model score. The median survival time of the group of patients predicted more sarcomatoid was significantly shorter compared with the lower scoring group of patients (190 vs. 402 days, $p < 0.002$). This difference in survival can be observed in the Kaplan Meier plot (Figure 6A), where the predicted non-sarcomatoid curve in orange is less steep than the blue predicted sarcomatoid curve. In a Cox-proportional hazard model adjusted for gender and age at diagnosis, the hazard ratio for sarcomatoid cases was 2.43 (95% confidence interval [CI] 1.44–4.12, $p < 0.005$), indicating that patients with sarcomatoid morphology were 2.43 times more likely to have died at a specific time point than non-sarcomatoid subjects. In comparison, the hazard ratio (HR) for both gender and age were both much smaller, at < 1.1. Very similar findings were obtained with censoring at 3 years (see the supplemental information; Figure 2).

DISCUSSION

We have developed a model capable of learning a cell-level indication of sarcomatoid and epithelioid regions of a TMA core tissue sample, which enables quantitative characterization of a core according to the relative proportions of S and E components present. In summary:

(1) We have developed a GNN model (called MesoGraph) that can predict the mesothelioma subtype of the given patient sample with high accuracy (AUROC > 0.85) over independent multicentric validation using only H&E-stained images of tumor samples.

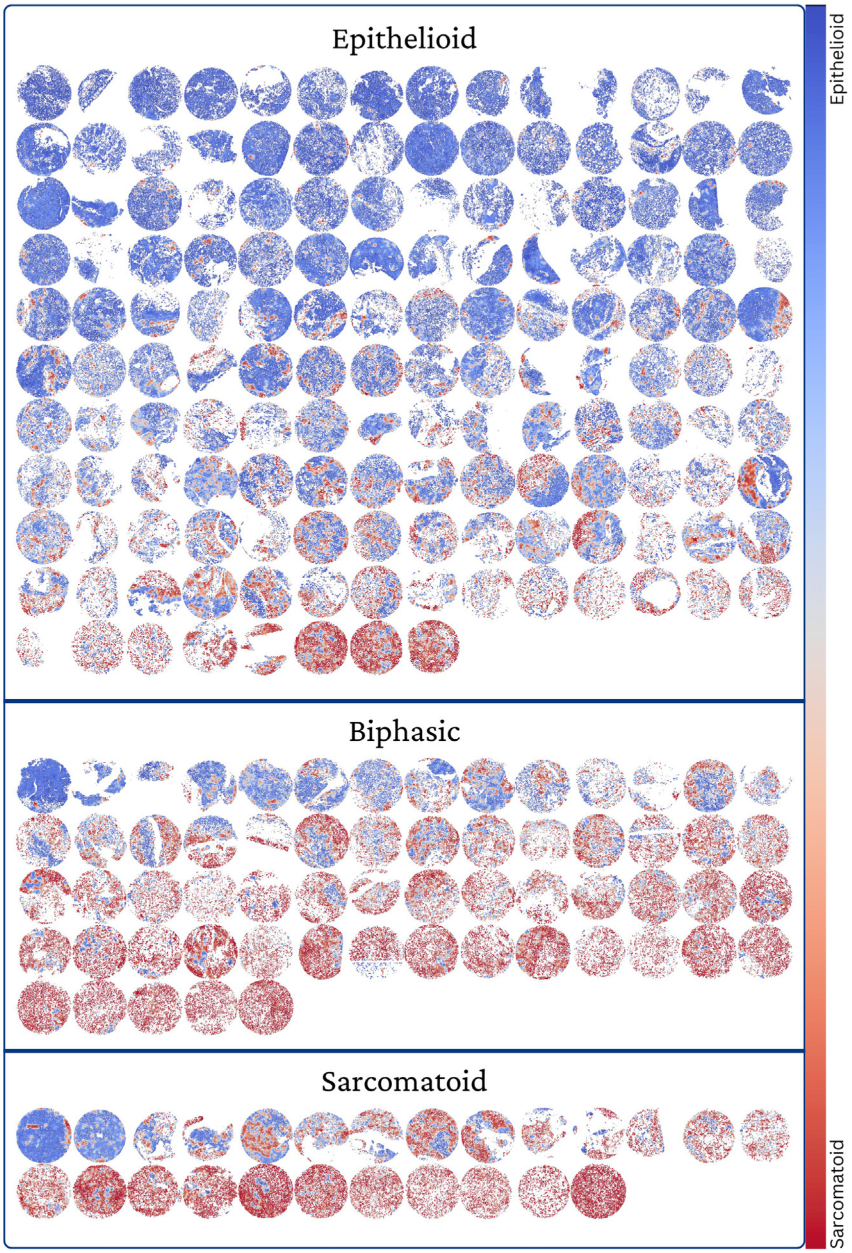

Figure 3. Overview of model predictions by subtype

Images of model predictions ordered within each subtype by the predicted predominance of the sarcomatoid component, illustrating the underlying continuous biological expression of tissue on the epithelioid to sarcomatoid spectrum.

(2) For a given sample, the proposed approach can generate a quantitative assessment (called MesoScore) of where the sample stands are in terms of the epithelioid to sarcomatoid spectrum.
(3) Model predictions can be mapped onto individual cells in a given sample to generate histograms (called MesoGrams) showing the relative densities of epithelioid and sarcomatoid components within the tumor.
(4) MesoGraph-generated scores can be used as a prognostic marker for predicting disease specific survival.
(5) We show that the weakly supervised model is able to characterize known morphological patterns of cells associated with EM and SM.
(6) The code and the dataset used in this study have been made publicly available for further development at https://github.com/measty/MesoGraph.

The developed approach could help pathologists to subtype a core more accurately, consistently, and efficiently and paves the way to move beyond the three-type system of characterizing a tumor toward a more fine-grained characterization that matches the underlying continuous biological expression of mesothelial tumor cells on a spectrum between epithelioid and sarcomatoid morphology.

Most MIL-based methods introduced in the literature have been patch based. One such MIL approach was introduced in Li et al.[17] Here, a dual-stream approach was used where the final bag score is the mean of max instance pooling and an attention-based weighted average of instances attended to by the max instance. In another approach,[18] large-scale datasets are used to train an MIL model for tumor detection, backpropagating only the top K instances per bag. The CLAM algorithm[14] is a further patch-based MIL method with attention that has been applied to a variety of computational pathology tasks. As a final example of patch-based approaches, in the IDaRS algorithm proposed in Bilal et al.[19] to detect key mutations on colorectal cancer, learning occurs on patches drawn using a ranking-based sampling scheme. While these MIL approaches have been developed for patch-level instances, we develop our method by treating each cell as an instance, allowing us to investigate the differences in cell morphology between subtypes. This also removes the limitation on spatial resolution of predictions imposed by a patch-based approach.

GNNs have also been applied in this domain. In Lu et al.,[20] a GNN is used on prostate cancer TMA cores with self-supervised and morphological features for the task of classifying examples as high or low risk according to the Gleason score. GNNs are applied to WSIs in Lu et al.[21] by spatially clustering cells to form agglomerate nodes from which to build a slide-level graph to predict HER2 status in breast cancer. Our approach uses a

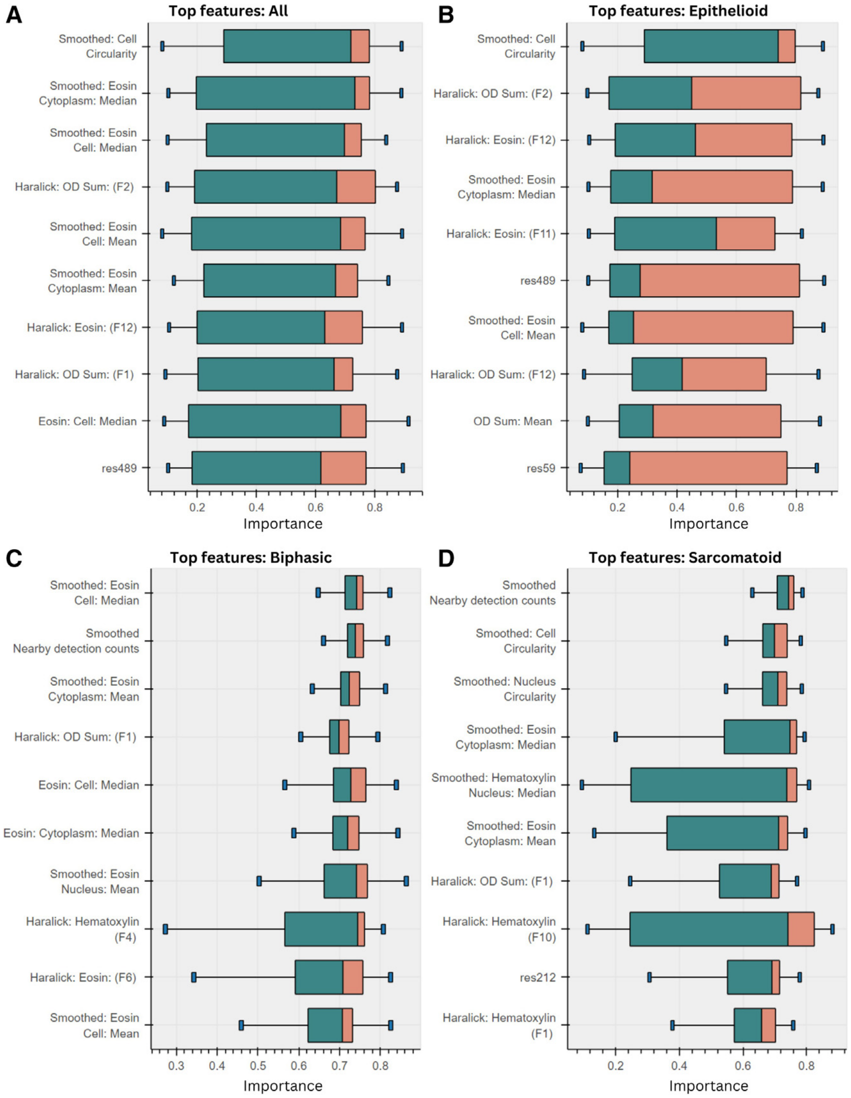

Figure 4. Illustration of the top 10 features identified by GNNExplainer

(A–D) considering all cores (A), and in (B)–(D), importances on cores grouped by subtype. Results shown as a standard box and whisker plot, with the box showing the 25th, 50th, and 75th percentile of a features importance scores over cores. Whiskers show min and max values, limited at box ± 1.5× inter-quartile range. The top feature is circularity, a known differentiating characteristic between mesothelial subtypes, providing validation for our model.

dual-task formulation with ranking loss, on a cell graph, to allow better identification of regions associated with the two components that may be present in a mesothelioma core.

The results for this study show a potential for clinical implications when applied to routine diagnosis of MM. As shown in our work, there is a gradient between epithelioid and sarcomatoid MM, and the various cell populations are identifiable and can be quantified using our approach. Improving identification of mesothelioma subtypes is an essential part of diagnosis for MM. The behavior of biphasic MM is dependent on the ratio of epithelioid and sarcomatoid cells and may also be extended to other biphasic tumors. The survival of BM is suspected to correlate with the amount of the sarcomatoid component.[22,23] The criteria for a sarcomatoid component are not well defined, and the interobserver variability between expert pathologists for identifying this component is moderate.[24] With the increasing use of digital pathology, this model represents a first point of entry for an AI-based clinical tool that can be applied by pathologists to more accurately define epithelioid and sarcomatoid components in mesothelioma, especially in BM, and potentially create future opportunities to improve clinical decision-making and prognosis. Diagnosis by pathologists of subtype and percentage of sarcomatoid and epithelioid components can guide treatment pathways.[23,25] Surgery and multimodality chemoradiation therapy are the most common treatments for all types of mesothelioma.[26,27] There are mixed results using chemotherapy and radiation for BM, and surgical therapies are largely ineffective for SM. However, the therapies have shown some success in extending life expectancy for epithelioid-type mesothelioma. Additionally, EM, and to lesser extent SM, has been shown to respond well to immune checkpoint inhibitors.[28] MesoGraph is a first-of-its-kind tool that allows for a precise and accurate determination of the fraction of sarcomatoid-type tumor cells. As such, MesoGraph has the potential to guide treatment options such as surgery and multimodality therapy options in a more precise manner, given that patients may be less responsive to therapy depending on the fraction of SM. Additionally, a precise determination of the fractions of epithelioid and sarcomatoid cells may assist in a more accurate assessment of individual patient prognosis.

One limitation of our method is that while we have taken care to validate our model by looking at the features that influence its predictions and the typical morphology of cells found in epithelioid and sarcomatoid regions, we still have some issues with the interpretability of the model outputs. Not all of the features our model learns on have an obvious histomorphological counterpart. For example, if we see from a feature importance analysis that a particular resnet feature is important, it is not clear how that translates into a histomorphological biomarker that a pathologist can look for in a tissue sample. Haralick texture features are a little better, as they are constructed to capture specific, well-defined properties of textures, but they are still difficult to interpret in comparison to morphological features.

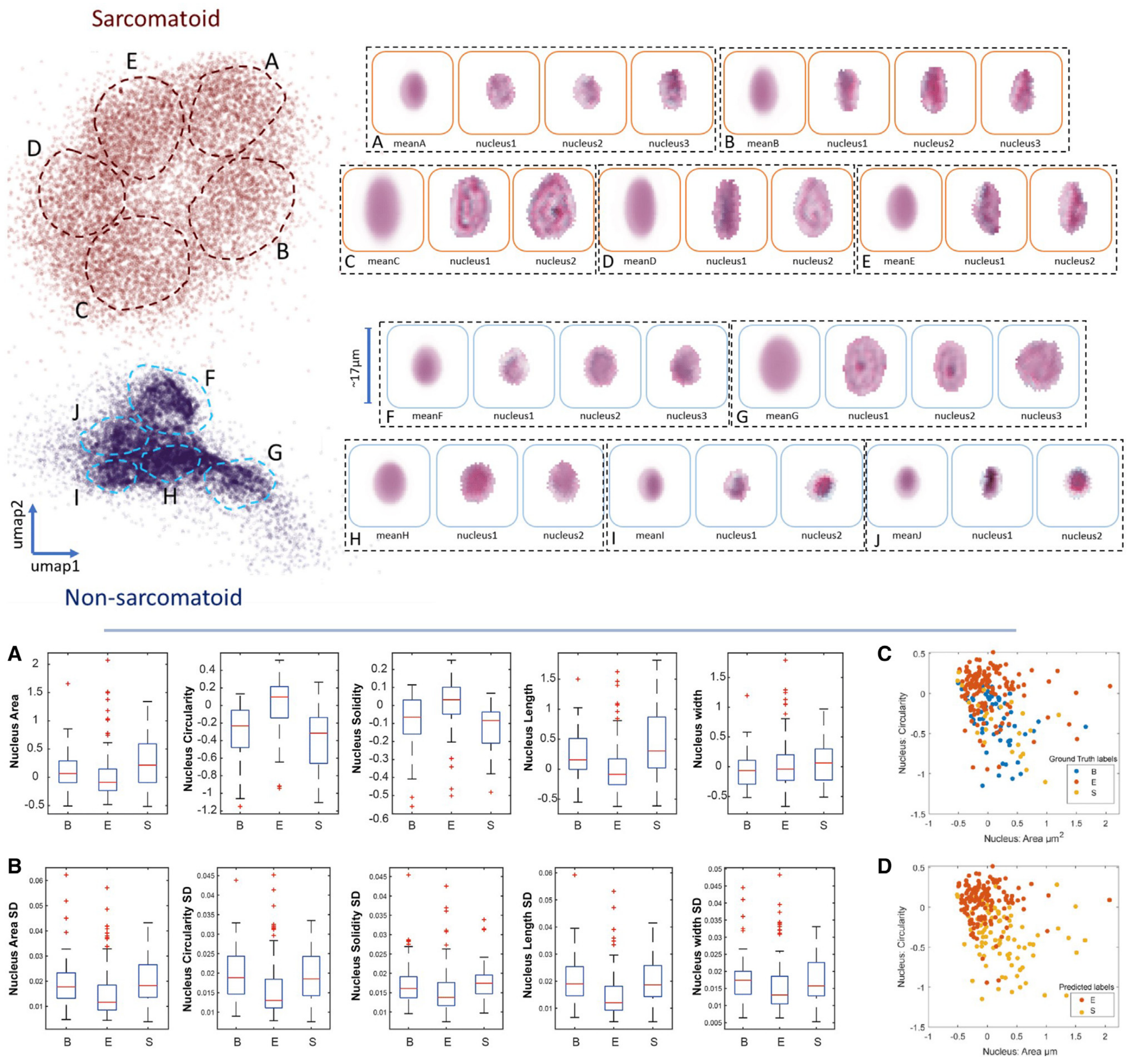

Figure 5. Illustrations of morphological differences between predicted subtypes

Top: examples of cells scored most and least highly by the model, plotted as a 2D UMAP reduction of principal components calculated on both high- and low-scoring cells. For each cluster, the mean of the cells is displayed, together with individual example cells. Clusters A–E, on the left, are predicted to be sarcomatoid and demonstrate a more spindle-like morphology, grouped together in size and relative spindle cell characteristics in each cluster as shown by example cells on the right. Similarly, the non-sarcomatoid predicted cells also show clustering into 5 groups, F–J. Bottom: morphological heterogeneity of mesothelioma tumors independent of model prediction.

(A) Distribution of average morphology across tumor types. All measurements are normalized to the data average and standard deviation.

(B) Heterogeneity of cell morphology across different mesothelioma tumor types based on standard deviation (SD) of *Z* scored single-cell data.

(C and D) Morphological heterogeneity based on ground-truth labels (C) and predicted labels (D).

Our use of TMA cores is also an area for improvement. The aim of the model is to define the epithelioid and sarcomatoid components of mesothelioma. By using TMAs that have preselected areas of tumor cells as defined by expert histopathologists, we increase the likelihood that we are training on mesothelioma tumor cells. The limitation of TMAs is that they are also highly selective because of being only representative of the tumor cell population, and in contrast to resection material, TMAs have only limited or very little additional surrounding tissue that will include spatial heterogeneity of tumor cells and

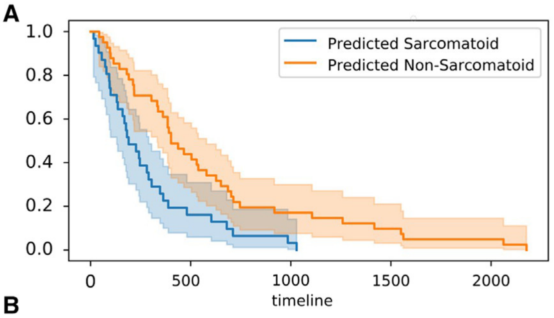

B

**Predicted Non-Sarcomatoid**					
At risk	41	18	7	4	2
Censored	0	0	0	0	0
Events	0	23	34	37	39
**Predicted Sarcomatoid**					
At risk	31	5	1	0	0
Censored	0	0	0	0	0
Events	0	26	30	31	31

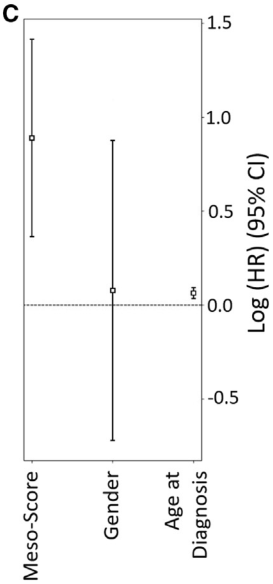

Figure 6. Survival prediction using Meso-Score

(A) Kaplan-Meier curves for all data. For data right censored at 3 years (see Figure S1).

(B) Cumulative events and total number at risk at each of the times shown on the x axis

(C) Log hazard ratio of high MesoScore compared to demographic factors.

microenvironment. We have illustrated our model output on regions of interest (ROIs) of a small number of biphasic slides from TCGA dataset, shown in Figure S3. We aim in further studies to modify our pipeline to use whole slides of resection material to further validate our model and expand on the role of the tumor microenvironment.

Future work could involve the incorporation of cell classifications via a segmentation method such as Hovernet,[29] capable of simultaneous cell segmentation and classification. This would provide a further informative feature that may help identify cell-type-specific patterns such as an association of tumor-infiltrating lymphocytes to a specific subtype. Such features could also help move away from difficult-to-interpret features such as resnet features, without sacrificing performance. A more extensive evaluation considering a larger dataset and including pathologist concordance studies to identify whether pathologists using such a tool would make more consistent and more accurate subtyping could also be considered.

In conclusion, we provide a method for more precisely characterizing epithelioid and sarcomatoid cell subtypes in a quantifiable and reproducible way. Given the importance of sarcomatoid subtypes for prognosis and deciding on treatment pathway, our method may potentially offer clinical implications for patient care. Improved subtyping of MM allows for gains in both the efficiency and reliability of assessment of mesothelioma tumor cell classification by a reporting pathologist. The method we present and future work using our approach to further define the epithelioid and sarcomatoid spectrum of MM may ultimately form a basis for improving treatment and prognosis for the patient.

Limitations of the study

There are two main limitations of our work. One limitation is the interpretability of the model outputs. While we have taken care to validate our model by looking at the features that influence its predictions and the typical morphology of cells found in epithelioid and sarcomatoid regions, not all of the features our model learns on have an obvious histomorphological counterpart, and these can be difficult to interpret in comparison to morphological features.

Our use of TMA cores is also a limitation. We have illustrated our model output on ROIs of a small number of biphasic slides from TCGA dataset, shown in Figure S3. However, in order to be most effective on WSIs, a modified pipeline trained on a large dataset of mesothelioma WSIs would be preferable.

STAR★METHODS

Detailed methods are provided in the online version of this paper and include the following:

- KEY RESOURCES TABLE
- RESOURCE AVAILABILITY
  - Lead contact
  - Materials availability
  - Data and code availability
- EXPERIMENTAL MODEL AND SUBJECT DETAIL
- METHOD DETAILS
  - Problem formulation
  - Building graph neural networks on tissue cores
  - GNN model architecture
  - Cell morphology characterization
  - Model performance and evaluation
- QUANTIFICATION AND STATISTICAL ANALYSIS

SUPPLEMENTAL INFORMATION

Supplemental information can be found online at https://doi.org/10.1016/j.xcrm.2023.101226.

ACKNOWLEDGMENTS

This project was funded by the CRUK-STFC Early Detection Innovation Award. F.M. and M.E. also acknowledge funding support from EPSRC grant EP/W02909X/1. We are grateful to The London Asbestos Support Awareness Group (https://www.lasag.org.uk/), the National Mesothelioma Virtual Bank (http://www.mesotissue.org/), and MesoBank UK (http://www.mesobank.com/) for their support. Material used in the research program was obtained from Mesobank, a Research Tissue Bank supported by Asthma and Lung UK and the Victor Dahdaleh Foundation. This work uses data that have been

provided by patients and collected by the NHS as part of their care and support. The data are collated, maintained, and quality assured by the National Cancer Registration and Analysis Service, which is part of NHS Digital. Access to the data was facilitated by the UK Health Security Agency Office for Data Release.

AUTHOR CONTRIBUTIONS

Conceptualization, M.E., F.M., J.O., J.L.R., H.S., S.T.M., X.G., and E.K.; lead for machine learning model and experiment design, F.M.; machine learning model design and implementation, M.E.; analysis, M.E. and F.M.; paper write-up, M.E., F.M., J.O., J.L.R., H.S., S.T.M., and X.G.; paper revisions, J.O., H.S., S.T.M., X.G., and E.K.; paper structuring, E.K.; funding acquisition, F.M., J.L.R., and H.S.; project supervision and management, F.M. and J.L.R.; statistical analysis, J.O.; clinical lead, J.L.R.; study design, H.S.; result analysis, H.S.; data provision, A.M.F., D.J., W.C., M.M., and S.P. F.M. and J.L.R. are joint last authors.

DECLARATION OF INTERESTS

The authors declare no competing interests.

INCLUSION AND DIVERSITY

We support inclusive, diverse, and equitable conduct of research.

Received: January 12, 2023
Revised: June 8, 2023
Accepted: September 14, 2023
Published: October 9, 2023

REFERENCES

1. Wagner, J.C., Sleggs, C.A., and Marchand, P. (1960). Diffuse pleural mesothelioma and asbestos exposure in the North Western Cape Province. Br. J. Ind. Med. *17*, 260–271.
2. Hjerpe A, D.K., and Abd-Own, S. (2018). Cytopathologic diagnosis of epithelioid and mixed-type malignant mesothelioma: Ten years of clinical experience in relation to international guidelines. Arch. Pathol. Lab Med. *142*, 893–901. https://doi.org/10.5858/arpa.2018-0020-RA.
3. Cancer Research UK. Mesothelioma statistics. https://www.cancerresearchuk.org/health-professional/cancer-statistics/statistics-by-cancer-type/mesothelioma.
4. Ai, J., and Stevenson, J.P. (2014). Current issues in malignant pleural mesothelioma evaluation and management. Oncol. *19*, 975–984. https://doi.org/10.1634/theoncologist.2014-0122.
5. Meyerhoff, R.R., Yang, C.-F.J., Speicher, P.J., Gulack, B.C., Hartwig, M.G., D'amico, T.A., Harpole, D.H., and Berry, M.F. (2015). Impact of mesothelioma histologic subtype on outcomes in the surveillance, epidemiology, and end results database. J. Surg. Res. *196*, 23–32.
6. Mansfield, A.S., Symanowski, J.T., and Peikert, T. (2014). Systematic review of response rates of sarcomatoid malignant pleural mesotheliomas in clinical trials. Lung Cancer *86*, 133–136.
7. WHO Classification of Tumours Editorial Board (2021). Thoracic Tumours. WHO Classification of Tumours, 5th Edition, *Volume 5*.
8. Courtiol, P., Maussion, C., Moarii, M., Pronier, E., Pilcer, S., Sefta, M., Manceron, P., Toldo, S., Zaslavskiy, M., Le Stang, N., et al. (2019). Deep learning-based classification of mesothelioma improves prediction of patient outcome. Nat. Med. *25*, 1519–1525.
9. Dacic, S., Le Stang, N., Husain, A., Weynand, B., Beasley, M.B., Butnor, K., Chapel, D., Gibbs, A., Klebe, S., Lantuejoul, S., et al. (2020). Interobserver variation in the assessment of the sarcomatoid and transitional components in biphasic mesotheliomas. Mod. Pathol. *33*, 255–262.
10. Naso, J.R., Levine, A.B., Farahani, H., Chirieac, L.R., Dacic, S., Wright, J.L., Lai, C., Yang, H.M., Jones, S.J.M., Bashashati, A., et al. (2021). Deep-learning based classification distinguishes sarcomatoid malignant mesotheliomas from benign spindle cell mesothelial proliferations. Mod. Pathol. *34*, 2028–2035. https://doi.org/10.1038/s41379-021-00850-6.
11. Courtiol, P., Tramel, E.W., Sanselme, M., and Wainrib, G. (2018). Classification and disease localization in histopathology using only global labels: A weakly-supervised approach. Preprint at arXiv. https://doi.org/10.48550/arXiv.1802.02212.
12. Durand, T., Thome, N., and Cord, M. (2016). Weldon: Weakly supervised learning of deep convolutional neural networks. In IEEE Conference on Computer Vision and Pattern Recognition (CVPR), pp. 4743–4752. https://doi.org/10.1109/CVPR.2016.513.
13. Eastwood, M., Marc, S., Gao, X., Sailem, H., Offman, J., Karteris, E., Fernandez, A., Jonigk, D., Cookson, W., Moffatt, M., et al. (2022). Malignant Mesothelioma Subtyping of Tissue Images via Sampling Driven Multiple Instance Prediction. In Artificial Intelligence in Medicine: 20th International Conference on Artificial Intelligence in Medicine, M. Michalowski, S.S.R. Abidi, and S. Abidi, eds. (AIME), pp. 263–272. https://doi.org/10.1007/978-3-031-09342-5_25.
14. Lu, M.Y., Williamson, D.F.K., Chen, T.Y., Chen, R.J., Barbieri, M., and Mahmood, F. (2020). Data efficient and weakly supervised computational pathology on whole slide images. Preprint at arXiv. https://doi.org/10.1048550/arXiv.2004.09666.
15. Ying, R., Bourgeois, D., You, J., Zitnik, M., Leskovec, J., and Gnnexplainer. (2019). Generating explanations for graph neural networks. Preprint at arXiv. https://doi.org/10.1048550/arXiv.1903.03894.
16. McInnes, L., Healy, J., Saul, N., and Großberger, L. (2018). UMAP: Uniform Manifold Approximation and Projection. J. Open Source Softw. *3*, 861. https://doi.org/10.21105/joss.00861.
17. Li, B., Li, Y., and Eliceiri, K.W. (2021). Dual-stream multiple instance learning network for whole slide image classification with self-supervised contrastive learning. In Proceedings of the IEEE/CVF Conference on Computer Vision and Pattern Recognition (CVPR), pp. 14318–14328.
18. Campanella, G., Hanna, M.G., Geneslaw, L., Miraflor, A., Werneck Krauss Silva, V., Busam, K.J., Brogi, E., Reuter, V.E., Klimstra, D.S., and Fuchs, T.J. (2019). Clinical-grade computational pathology using weakly supervised deep learning on whole slide images. Nat. Med. *25*, 1301–1309.
19. Bilal, M., Raza, S.E.A., Azam, A., et al. (2021). Novel deep learning algorithm predicts the status of molecular pathways and key mutations in colorectal cancer from routine histology images. Preprint at medRxiv. https://doi.org/10.1101/2021.01.19.21250122.
20. Wang, J., Chen, R.J., Lu, M.Y., Baras, A., and Mahmood, F. (2019). Weakly Supervised Prostate TMA Classification via Graph Convolutional Networks. Preprint at arXiv. https://doi.org/10.1048550/arXiv.1910.13328.
21. Lu, W., Toss, M., Rakha, E., Rajpoot, N., and Minhas, F. (2021). Slide-graph+: Whole slide image level graphs to predict her2status in breast cancer. Preprint at. https://doi.org/10.1048550/arXiv:2110.06042.
22. Travis, W., Brambilla, E., Burke, A., Marx, A., and Nicholson, A. (2015). WHO Classification of Tumors of the Lung, Pleura, Thymus and Heart (IARC Press).
23. Galateau-Salle, F., Churg, A., Roggli, V., and Travis, W.D.; World Health Organization Committee for Tumors of the Pleura (2016). The 2015 World Health Organization Classification of Tumors of the Pleura: Advances since the 2004 Classification. J. Thorac. Oncol. *11*, 142–154.
24. Galateau Salle, F., Le Stang, N., Nicholson, A.G., Pissaloux, D., Churg, A., Klebe, S., Roggli, V.L., Tazelaar, H.D., Vignaud, J.M., Attanoos, R., et al. (2018). New Insights on Diagnostic Reproducibility of Biphasic Mesotheliomas: A Multi-Institutional Evaluation by the International Mesothelioma Panel From the MESOPATH Reference Center. J. Thorac. Oncol. *13*, 1189–1203.
25. Travis, W.D., Brambilla, E., Burke, A.P., Marx, A., and Nicholson, A.G. (2015). Introduction to the 2015 World Health Organization Classification of Tumors of the Lung, Pleura, Thymus, and Heart. J. Thorac. Oncol. *10*, 1240–1242.

CellPress
OPEN ACCESS

26. Kelly, R.J., Sharon, E., and Hassan, R. (2011). Chemotherapy and targeted therapies for unresectable malignant mesothelioma. Lung Cancer *73*, 256–263.

27. Santoro, A., O'Brien, M., Stahel, R.A., Nackaerts, K., Baas, P., Karthaus, M., Eberhardt, W., Paz-Ares, L., Sundstrom, S., Liu, Y., et al. (2008). Pemetrexed plus cisplatin or pemetrexed plus carboplatin for chemonaïve patients with malignant pleural mesothelioma: results of the International Expanded Access Program. J. Thorac. Oncol. *3*, 756–763.

28. Alley, E.W., Lopez, J., Santoro, A., Morosky, A., Saraf, S., Piperdi, B., and van Brummelen, E. (2017). Clinical safety and activity of pembrolizumab in patients with malignant pleural mesothelioma (keynote-028): preliminary results from a non-randomised, open-label, phase 1b trial. Lancet Oncol. *18*, 623–630.

29. Graham, S., Vu, Q.D., Raza, S.E.A., Azam, A., Tsang, Y.W., Kwak, J.T., and Rajpoot, N. (2019). Hover-Net: Simultaneous segmentation and classification of nuclei in multi-tissue histology images. Med. Image Anal. *58*, 101563.

30. Rintoul, R.C., Rassl, D.M., Gittins, J., and Marciniak, S.J.; MesobanK collaborators (2016). MesobanK UK: an international mesothelioma bioresource. Thorax *71*, 380–382. https://doi.org/10.1136/thoraxjnl-2015-207496.

31. Vahadane, A., Peng, T., Sethi, A., Albarqouni, S., Wang, L., Baust, M., Steiger, K., Schlitter, A.M., Esposito, I., and Navab, N. (2016). Structure-preserving color normalization and sparse stain separation for histological images. IEEE Trans. Med. Imag. *35*, 1962–1971.

32. Dietterich, T.G., Lathrop, R.H., and Lozano-Pérez, T. (1997). Solving the multiple instance problem with axis-parallel rectangles. Artif. Intell. *89*, 31–71. https://doi.org/10.1016/S0004-3702(96)00034-3.

33. Schmidt, U., Weigert, M., Broaddus, C., and Myers, G. (2018). Cell detection with star-convex polygons. In Medical Image Computing and Computer Assisted Intervention - MICCAI 2018, A. Frangi, J. Schnabel, C. Davatzikos, C. Alberola-López, and G. Fichtinger, eds. (Springer), pp. 265–273.

34. Bankhead, P., Loughrey, M.B., Fernández, J.A., Dombrowski, Y., McArt, D.G., Dunne, P.D., McQuaid, S., Gray, R.T., Murray, L.J., Coleman, H.G., et al. (2017). QuPath: Open source software for digital pathology image analysis. Sci. Rep. *7*, 1.16878. https://doi.org/10.1038/s41598-017-17204-5.

35. Haralick, R.M., Shanmugam, K., and Dinstein, I. (1973). Textural features for image classification. IEEE Trans. Syst. Man Cybern. *3*, 610–621. https://doi.org/10.1109/TSMC.1973.4309314.

36. Xu, K., Hu, W., Leskovec, J., and Jegelka, S. (2018). How Powerful are Graph Neural Networks?. Preprint at. https://doi.org/10.1048550/arXiv:1810.00826.

37. Wang, Y., Sun, Y., Liu, Z., Sarma, S.E., Bronstein, M.M., and Solomon, J.M. (2018). Dynamic Graph CNN for Learning on Point Clouds. Preprint at arXiv. https://doi.org/10.1048550/arXiv:1801.07829.

38. Xu, K., Li, C., Tian, Y., Sonobe, T., Kawarabayashi, K.-i., and Jegelka, S. (2018). Representation learning on graphs with jumping knowledge networks. In Proceedings of the 35th International Conference on Machine Learning, J. Dy and A. Krause, eds. (PMLR), pp. 5453–5462.

39. Kingma, D.P., Ba, J., and Adam. (2017). A method for stochastic optimization. Preprint at arXiv. https://doi.org/10.48550/arXiv:1412.6980.

40. Smith, L.N. (2015). Cyclical learning rates for training neural networks. Preprint at arXiv. https://doi.org/10.48550/arXiv:1506.01186.

41. Turk, M., and Pentland, A. (1991). Face recognition using eigenfaces. In Proceedings of 1991 IEEE Computer Society Conference on Computer Vision and Pattern Recognition (IEEE), pp. 586–591. https://doi.org/10.1109/CVPR.1991.139758.

STAR★METHODS

KEY RESOURCES TABLE

REAGENT or RESOURCE	SOURCE	IDENTIFIER
Deposited data		
Mesobank	https://www.mesobank.com/	meso TMA dataset
Software and algorithms		
tiatoolbox	https://github.com/TissueImageAnalytics/tiatoolbox/	v1.4
torch geometric	https://pytorch-geometric.readthedocs.io/en/latest/install/installation.html	v2.3
bokeh	http://bokeh.org/	v3.1
lifelines	https://lifelines.readthedocs.io/en/latest/	v0.25.10
QuPath	https://doi.org/10.1038/s41598-017-17204-5	v0.3.0
GNNExplainer	https://arxiv.org/abs/1903.03894	v2.3 (part of torch-geometric)
torch geometric	https://pytorch-geometric.readthedocs.io/en/latest/install/installation.html	v2.3
Original MesoGraph code	https://github.com/measty/MesoGraph	original research code

RESOURCE AVAILABILITY

Lead contact

Further information and requests for resources and reagents should be directed to and will be fulfilled by the Lead Contact, Mark Eastwood (Mark.Eastwood@warwick.ac.uk).

Materials availability

This study did not generate new materials.

Data and code availability

- Tissue Micro-array cores and labels for the primary cohort are linked in the github repository at: https://github.com/measty/MesoGraph The Mesobank data is available from Mesobank (https://www.mesobank.com/) on request. This would require the completion of mesobank's standard application form. It would then be reviewed to make sure that the proposed use of the data is covered by mesobank's generic ethical approval, and a suitable Data Sharing Agreement would need to be in place before any data is released.
- All original code is publicly available at: https://github.com/measty/MesoGraph.
- Any additional data is available from the lead contact on request.

EXPERIMENTAL MODEL AND SUBJECT DETAIL

The project was run according to the Imperial Research Codes of Practice and in line with the funder's terms and conditions. Two independent MM patient cohorts were obtained retrospectively (see Figure 1A): The training cohort was from St. Georges Hospital and consisted of 102 patients. The validation cohort, of 82 patients, was obtained from Mesobank,[30] a UK mesothelioma biobank. Mesobank collects samples from multiple UK hospitals. The date of death for Mesobank patients had been provided by the UK National Cancer Registration and Analysis Service (NCRAS).

The primary dataset used in this work is a collection of H&E stained Tissue Micro-arrays (TMAs) of tumor tissue biopsies collected from St. Georges Hospital, London. It consists of 4 Tissue Micro-array (TMA) slides scanned using a Hamamatsu Nanozoomer S360 scanner at 20× (0.4415 microns per pixel) with a total of 279 cores covering 102 separate cases (patients). After the removal of dropped and severely damaged/incomplete cores, we are left with 234 cores over 90 patients, of which 148 are EM, 61 BM, and 25 SM. We additionally use a validation set of TMA cores over two slides provided by Mesobank, scanned at 20× (0.5015 microns per pixel) using a Leica Aperio AT2. The class counts after removal of dropped/damaged cores were 258 cores over 77 patients, with 155 EM, 68 BM, and 35 SM. Only core-level labels are available. We first perform Vahadane stain normalization[31] to minimize systematic stain variability between slides and cores. To represent a TMA core as a graph suitable for learning a GNN, we detect cells and extract features from these as described in Building graph neural networks on tissue cores.

METHOD DETAILS

Problem formulation

As the biphasic subtype is a mix of epithelioid and sarcomatoid components, and subtype labels are only available at the core level, we model the subtype prediction task as a binary Multiple Instance Learning (MIL) problem. Under the MIL paradigm,[32] an example is represented by a bag of instances, and a bag is considered positive if it contains at least one positive sample. We express the subtyping problem as a dual MIL prediction task. In the first task, SM is considered the positive instance, whereas in the second task EM is considered the positive instance. Formulating the problem as two parallel MIL tasks allows the possibility for some instances to be negative instances in both tasks, in contrast to viewing any instance that is not sarcomatoid as being epithelioid which would be implicitly assumed in any single-task MIL formulation.

The goal of a MIL predictor is to use training data consisting of bags with bag level labels only to predict both bag and instance level labels in testing. Formally, let $\mathcal{B} = \{X_1, \ldots, X_{n_\mathcal{B}}\}$ be a bag corresponding to a single TMA core in our dataset, where X_i are instances (cells) within the bag. The number of instances $n_\mathcal{B}$ can vary across bags. Each core, represented by bag $\mathcal{B}$, is associated with a label $Y_\mathcal{B} \in \{0, 1, 2\}$ in the training dataset. In our formulation, considering SM as the positive instance, epithelioid-labelled cores take the label ($Y_\mathcal{B} = 0$), and biphasic and sarcomatoid cores take the label ($Y_\mathcal{B} = 1, Y_\mathcal{B} = 2$) respectively, as we expect progressively more sarcomatoid instances in BM and SM examples. Conversely, in the dual task (where EM is considered the positive class), biphasic and epithelioid cores take the bag labels ($Y_\mathcal{B} = 1, Y_\mathcal{B} = 2$), with sarcomatoid becoming the negative example ($Y_\mathcal{B} = 0$). This labeling system, by predominance of positive instances, is a departure from that typically used in the MIL setting, where only positive ($Y_\mathcal{B} = 1$) and negative ($Y_\mathcal{B} = 0$) bags exist. We deal with this with our use of a ranking-based loss, as detailed in GNN model architecture. Our goal is then to build a machine learning model $F(\mathcal{B}; \Phi)$ with trainable parameters Φ that can use a labeled training dataset $D = \{(\mathcal{B}_1, Y_1), (\mathcal{B}_2, Y_2), \ldots, (\mathcal{B}_M, Y_M)\}$ to generate a predicted label for a test core $\mathcal{B}$. This is done by aggregating instance level predictions $z_i = g(X_i; \Phi)$ to give $Z_B = F(\mathcal{B}; \Phi) = Agg(\{z_i = g(X_i; \Phi) | X_i \in \mathcal{B}\})$ through an appropriate aggregation function $Agg(\cdot)$ such as max or average across top most positive instances.

Modeling the mesothelioma subtyping problem through MIL allows us to use core-level labels to learn an instance-level scoring, with which we can identify predominantly EM or SM regions in a core. This enables us to quantify where each tissue sample falls in the EM-to-SM continuum according to the relative proportions of SM and EM instances. This fine-grained and natural characterization of a tumor can lead to more informed decisions regarding treatment.

Building graph neural networks on tissue cores

A tissue sample can be described by its individual component cells and their spatial arrangement within the sample. Their physical proximity will result in nearby cells affecting each other, through their shared micro-environment and interaction via various biological processes. Therefore, a natural way to represent the sample is as a graph, with each cell being a node in the graph, connected to other nearby cells in its neighborhood. Let $G = (V, E)$ denote a graph, where V and E are the sets of nodes and edges respectively. Each node $v \in V$ is associated with a feature vector X_v. In our case, each node v is a cell, with features X_v describing characteristics of the cell and its immediate surroundings.

We use Stardist[33] within QuPath[34] to perform cell detection on the TMA cores. Stardist is an approach to cell detection which uses star-convex polyhedra to represent objects. For each pixel, the distances to the boundary of its containing object along a set number of radial directions are learned.

For each detected cell, we use QuPath to extract features describing both the cell, and the region surrounding it, including some haralick texture features as described in Haralick et al.[35] for a total of 157 features as described below.

- Shape features: Area, length, circularity, Max and Min diameter for both nucleus and whole cell
- Intensity features: Mean, Median and Standard Deviation for hematoxylin and eosin channels over cell nucleus, cell cytoplasm and whole cell
- Shape/intensity smoothed: Above features smoothed over nearby cells using a Gaussian kernel of diameter 50 μm
- Delaunay cluster features: number of neighbors, edge length statistics, cluster means of above features.
- Haralick texture features on a small circular region around detection: calculated on the eosin channel, the hematoxylin channel and on the OD sum.

In addition to these features, we extract 72×72 image patches centered at the centroid of each cell and use a resnet34 (imagenet pretrained weights) to extract a further 512 features for each cell, taken from the penultimate layer output of the resnet model. We then construct the graph by connecting cells to each other cell whose centroid lies within a small radius, which we set at 30 μm. The process of building the cell graph is illustrated in Figure 1B.

GNN model architecture

Graph neural networks (GNNs) are a powerful tool for representation learning on graphs. GNNs typically follow a neighborhood aggregation strategy,[36] where we update the representation of a node iteratively by a learned aggregation of the representations of its neighbors. To learn the dual MIL task as described in Problem formulation, our architecture branches after the neighborhood

aggregation layers. We denote the branches as Sarcomatoid (S) and Epithelioid (E) branch after the instances considered as positive in each task. An illustration of our GNN architecture can be found in Figure 1C.

Different GNN implementations vary in how they perform this aggregation, and how they combine the aggregation with the nodes current representation. We use the EdgeConv approach to aggregation from Wang et al.,[37] which at layer $k > 1$ takes the form:

$$h_v^{(k)} = \frac{1}{|N_v|} \sum_{u \in N_v} f_{\Theta_k} \left(h_v^{k-1} \| h_v^{(k-1)} - h_u^{(k-1)} \right)$$

here, N_v is the neighborhood of node v (i.e., the set of all nodes to which v is connected), $\|$ denotes concatenation, and f_{Θ_k} is chosen to be a multi-layer perceptron (MLP) with parameters Θ_k. The feature representation at each layer is $h_v^{(k)} \in \mathbf{R}^{d_k}$ and the initial representation of the node is the feature vector, $h_v^{(0)} = X_v \in \mathbf{R}^{d_0}$. The output of the first layer is a purely local transformation $h_v^{(1)} = f_{\Theta_1}(X_v)$, where again f_{Θ_1} is an MLP with parameters Θ_1. At each layer we choose f_{Θ_k} to be an MLP with one hidden layer, $\mathrm{MLP}(d_{k-1}, d_k)$ with input dimension d_{k-1} and hidden layer and output dimension d_k. Rather than computing the final output z_v at a node from the representation in the final layer only, we follow the concatenation approach in Jumping Knowledge Networks[38] to combine the representation at different layers.

This combined representation from the graph convolution layers is passed to the E and S branches, to give for the S branch:

$$z_v^{(s)} = \sigma\left(\alpha^{(s)} f_{\Theta_s} \left(\left[h_v^{(1)} \| \ldots \| h_v^{(K)} \right] \right) + \beta^{(s)} \right)$$

Here $\sigma(\cdot)$ denotes a sigmoid function, and both $\alpha^{(s)} = f_\alpha^{(s)}(\overline{X})$ and $\beta^{(s)} = f_\beta^{(s)}(\overline{X})$ are the output of further small MLPs taking as input a core-level feature mean $\overline{X} = \frac{1}{N} \sum_{v \in V} X_v$. In a similar way, we also obtain $z_v^{(e)}$ for the E branch. We take the graph level prediction to be $Z = \frac{1}{|V|} \sum_{v \in V} (z_v^{(s)} - z_v^{(e)})$, the mean of the cell-level scores.

To train our model, we use a pairwise ranking loss:

$$L = \sum_{i \in \mathrm{Batch}} \sum_{j \in \mathrm{Batch}} \max(0, 1 - (Y_i - Y_j)(Z_i - Z_j))$$

where one prediction head (treating S as the positive instance) is trained to rank bags $S > B > E$ and the second is trained to rank $E > B > S$, i.e., treating E as the positive instance. Our model is implemented using the PyTorch geometric framework. We used 5 EdgeConv layers, each learning a feature representation of dimension 10. We use the Adam optimiser[39] and a decaying cyclic learning rate scheme[40] with min and max learning rate 2×10^{-5} and 1×10^{-4}. The cycle length is 50 epochs and at each cycle, the max lr decays by a factor of 0.8. We train for a maximum of 500 epochs with early stopping.

Cell morphology characterization

To investigate the typical cell morphologies and morphological differences of cells assigned high and low scores by the model, our approach is similar in concept to the 'eigenfaces' decomposition in Turk and Pentland.[41] We have taken the highest scoring 10% of cells from sarcomatoid cores, and the lowest scoring 10% of cells from epithelioid cores, and aligned the images of all the cells so that the major axis is oriented vertically. We have then masked out all but a small region around the cell so that as little background as possible remains. Finally, we have taken the H channel of the aligned cell images, and performed Principal Component Analysis (PCA) on the pixel values. This process and some of the resulting components are illustrated in Figure S2.

We use this analysis to illustrate the differences in morphology between cells scored highly sarcomatoid or non-sarcomatoid by our model, as presented in Characterization of cellular morphologies.

Model performance and evaluation

For performance evaluation on the primary cohort, we employ a hold-one-out cross-validation strategy over slides, so that for each fold all cores of a single slide are held out as the test set. This is done to avoid any potential bias from systematic differences between slides, and to ensure no mixing of cores from the same patient occurs between the training and testing sets. The cores to be used for training are split 75%–25% into train and validation sets, respectively. We compared our model with CLAM,[14] and PINS,[13] two patch-based methods which attempt to focus training in an adaptive way on the most important instances. We additionally compared with two simple MIL approaches, max-MIL and naive-MIL. Max-MIL is a patch-based method where we backpropagate only on the maximal instance during training. This has been used in for example.[18] Naive-MIL is a naive approach whereby we simply assign the bag level label to all instances in a bag, and treat all instances equally during training. For both of these methods we used a resnet34 pre-trained on imagenet as the base patch level model. To evaluate performance on the external validation cohort, we have trained our model on the entire St. Georges cohort, and evaluated model predictions on the Mesobank cohort. Conversely, we also present results obtained training on Mesobank data and evaluate that model on the St. Georges data. Model performance is summarized in Table 1, and Figure S4 as described in Predictive performance.

CellPress
OPEN ACCESS

QUANTIFICATION AND STATISTICAL ANALYSIS

Survival analysis was done using lifelines in python. The log-rank test was used for p values and the Kaplan-Meier estimator was used for plotting the survival curves. Relevant details can be found in Survival analysis using MesoScores and in Figure 6.

Performance metric calculations found in Predictive performance, Figure S4, and Table 1 were done in python using scikit-learn. Center and dispersion definitions used were mean and standard deviation.

Relevant values of *n* were 234 for St. Georges dataset, 258 for mesobank dataset, where *n* is number of TMA core images.

No specific methods were used to determine if data met the assumptions of the statistical approach.

Cell Reports Medicine, Volume *4*

Supplemental information

MesoGraph: Automatic profiling of mesothelioma subtypes from histological images

Mark Eastwood, Heba Sailem, Silviu Tudor Marc, Xiaohong Gao, Judith Offman, Emmanouil Karteris, Angeles Montero Fernandez, Danny Jonigk, William Cookson, Miriam Moffatt, Sanjay Popat, Fayyaz Minhas, and Jan Lukas Robertus

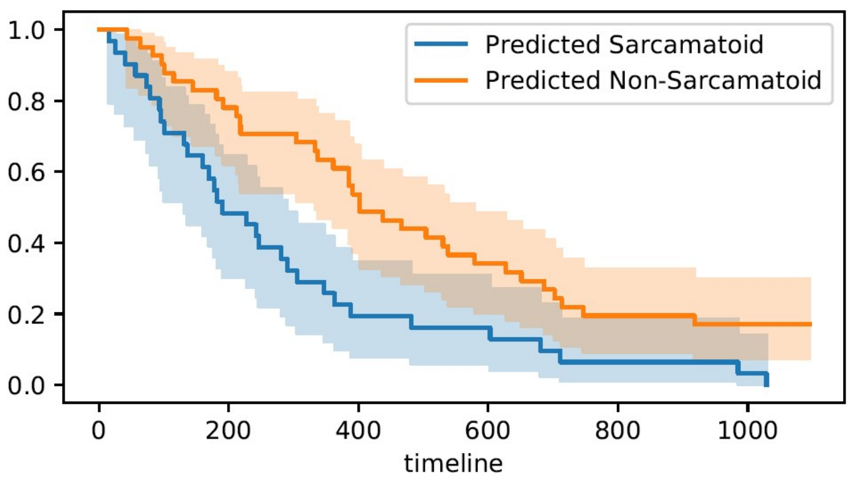

**Predicted Non-Sarcamatoid**						
At risk	41	32	22	14	8	7
Censored	0	0	0	0	0	0
Events	0	9	19	27	33	34
**Predicted Sarcamatoid**						
At risk	31	15	6	5	2	1
Censored	0	0	0	0	0	0
Events	0	16	25	26	29	30

Figure 1: Kaplan-Meier curves for survival prediction. Related to Figure 5. Shows curves when stratifying by model output score with data right censored at 3 years.

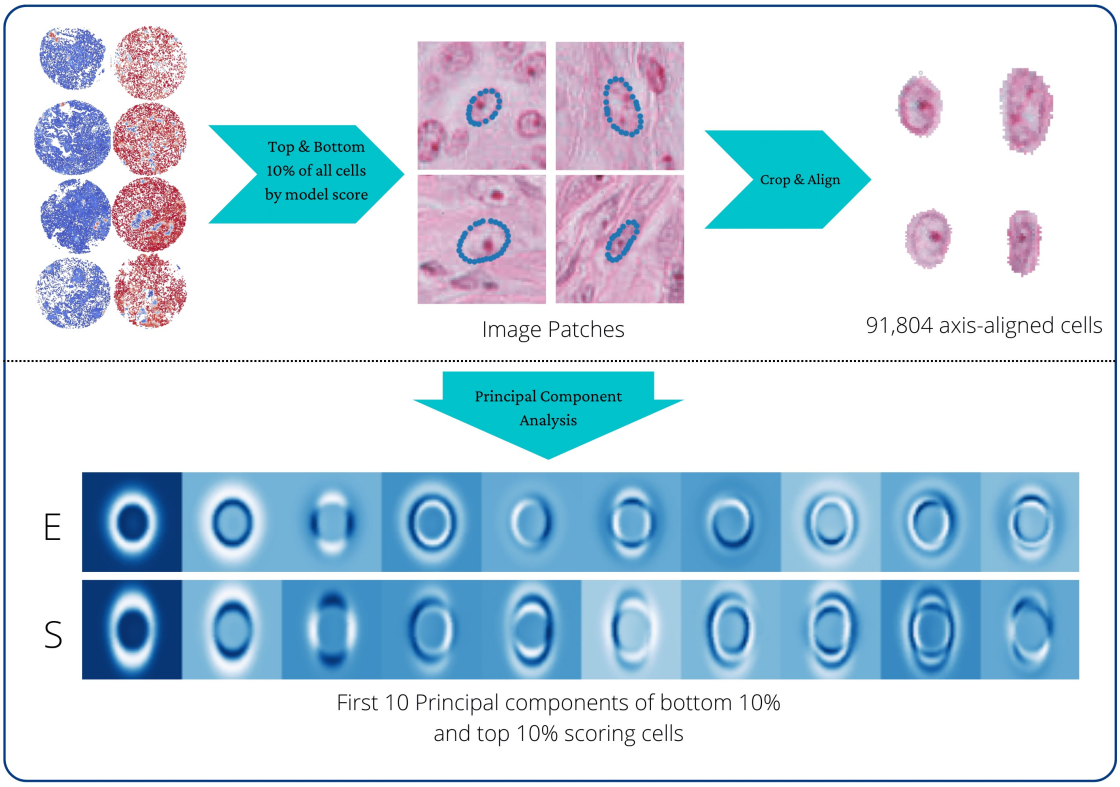

Figure 2: Principal component analysis on the top & bottom 10% of cells by model score. Related to Figure 4. Top and bottom scoring cell populations were oriented so that their major axes are oriented in the same direction, and cropped to remove as much background as possible. The resulting PCA components show highly sarcomatoid-scoring cells align with known morphological features of sarcomatoid subtype.

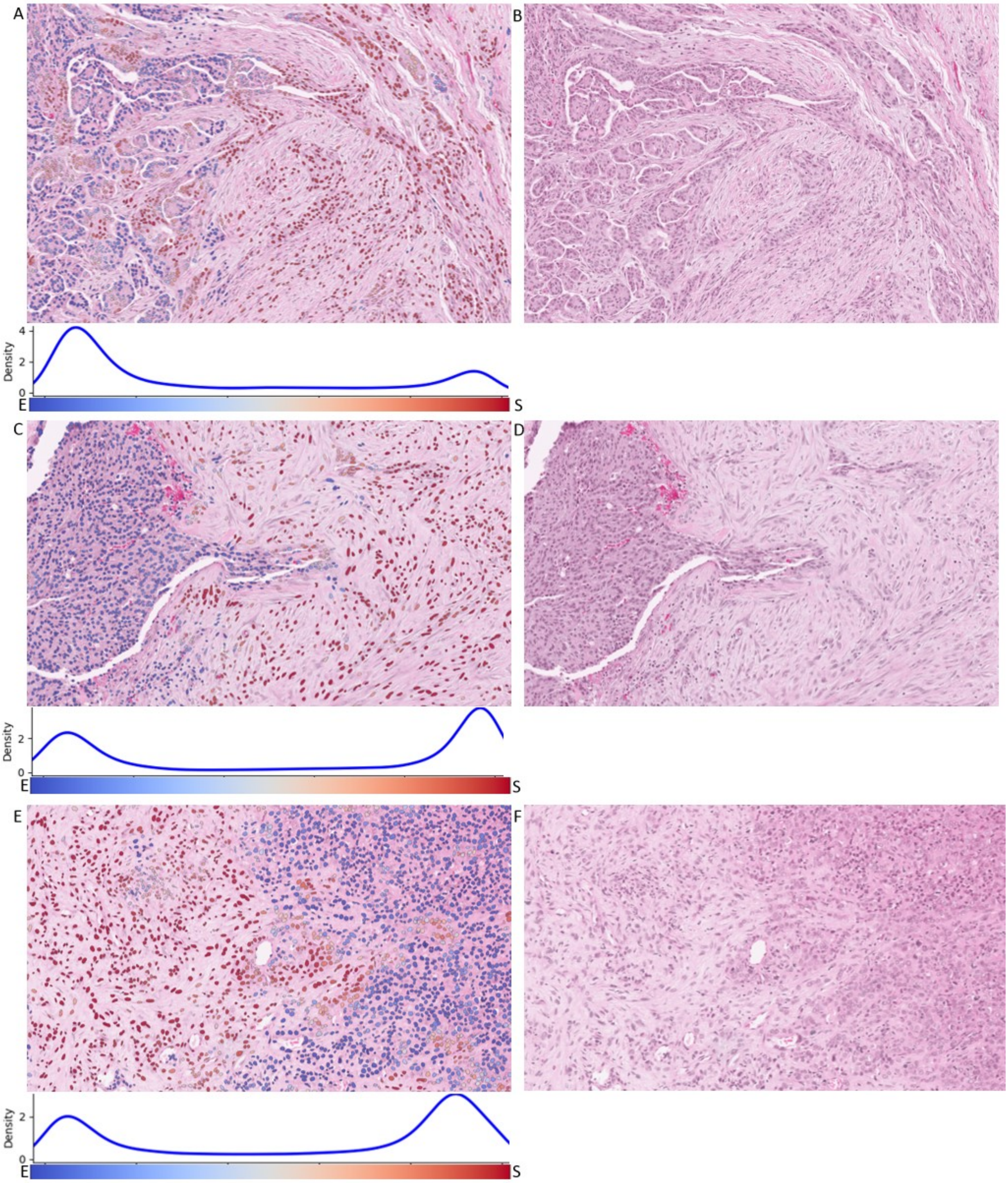

Figure 3: Model results on biphasic TCGA ROIs. Related to Figure 1. Illustrates the ability of MesoGraph to quantify regions of differing histological subtype. On the left in panels A, C and E are the rois overlaid with model output, and on the right in panels B, D and F are the raw ROI images. Distinct regions of sarcomatoid and non-sarcomatoid component can be seen defined by the model. The 'Mesogram' plot below each overlaid image shows the distribution of cell scores in the ROI.

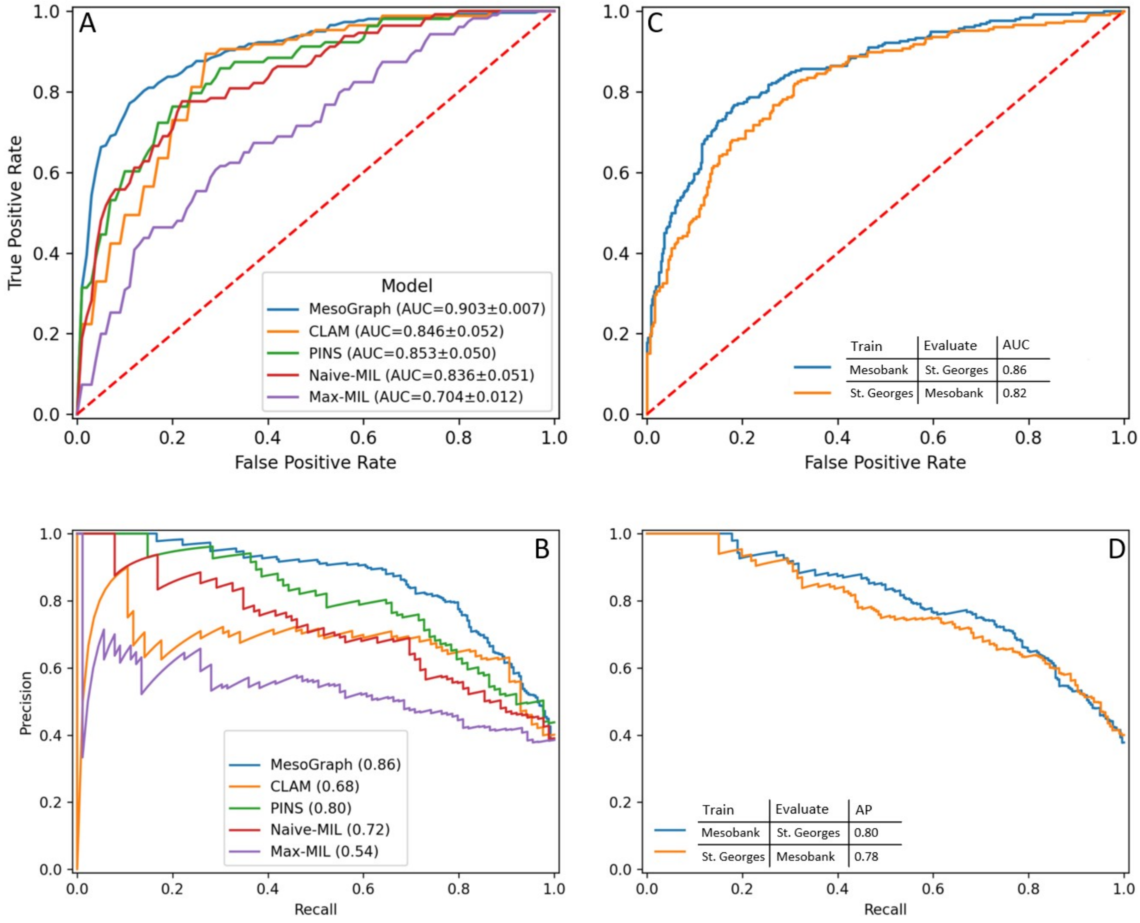

Figure 4: AUROC curves for experiments performed. Related to Table 1. A) AUROC for each model. B) Model average precision. C) AUROC on cross-cohort evaluation. D) Average precision on cross-cohort evaluation. Our model outperforms other models tested, and from C and D we see good generalization to external validation data.
